# Effect of amine structure on CO_2_ capture by polymeric membranes

**DOI:** 10.1080/14686996.2017.1399045

**Published:** 2017-11-22

**Authors:** Ikuo Taniguchi, Kae Kinugasa, Mariko Toyoda, Koki Minezaki

**Affiliations:** ^a^ International Institute for Carbon-Neutral Energy Research (WPI-I^2^CNER), Kyushu University, Fukuoka, Japan; ^b^ Graduate School of Integrated Frontier Sciences, Kyushu University, Fukuoka, Japan

**Keywords:** Amine structure, CO_2_, gas permeation, membrane, separation, 50 Energy Materials, 105 Low-Dimension (1D/2D) materials, 206 Energy conversion / transport / storage / recovery

## Abstract

Poly(amidoamine)s (PAMAMs) incorporated into a cross-linked poly(ethylene glycol) exhibited excellent CO_2_ separation properties over H_2_. However, the CO_2_ permeability should be increased for practical applications. Monoethanolamine (MEA) used as a CO_2_ determining agent in the current CO_2_ capture technology at demonstration scale was readily immobilized in poly(vinyl alcohol) (PVA) matrix by solvent casting of aqueous mixture of PVA and the amine. The resulting polymeric membranes can be self-standing with the thickness above 3 μm and the amine fraction less than 80 wt%. The gas permeation properties were examined at 40 °C and under 80% relative humidity. The CO_2_ separation performance increased with increase of the amine content in the polymeric membranes. When the amine fraction was 80 wt%, the CO_2_ permeability coefficient of MEA containing membrane was 604 barrer with CO_2_ selectivity of 58.5 over H_2_, which was much higher than the PAMAM membrane (83.7 barrer and 51.8, respectively) under the same operation conditions. On the other hand, ethylamine (EA) was also incorporated into PVA matrix to form a thin membrane. However, the resulting polymeric membranes exhibited slight CO_2_-selective gas permeation properties. The hydroxyl group of MEA was crucial for high CO_2_ separation performance.

## Graphical abstract

**Figure:**
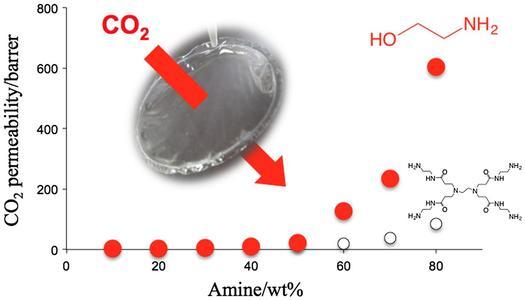

## 1. Introduction

CO_2_ is the major component of greenhouse gases (GHGs), which cause the global warming and climate change. Addressing the GHG issue, the Paris agreement has warned to limit a global temperature rise to 1.5 °C [1], and mitigation of the GHG emission is an urgent task to be tackled. Carbon capture and storage (CCS) has been approved as one of the effective solutions toward the issue, and about 20 full-scale CCS demonstrations have been planned or carried out all over the world [2]. In the CCS, CO_2_ is captured at the mass emission sources without exhausting to atmosphere, such as thermal power plants and steel works, and then injected into aquifer of certain geological structures underground or under seabed [3]. However, for implementation of the CCS, the cost reduction is inevitable, in which the CO_2_ capture dominates of the total. Liquid amine scrubbing is the most widely investigated CO_2_ capture method [4,5] and adopted in the large-scale demonstrations [2]. For example, CO_2_ in the flue gas from a coal-fired plant was captured by aqueous amine over other gaseous species, and then recovered by heating. The heating process is energy intensive even at the thermal power stations, which resulted in high cost. Thus, alternate low-energy CO_2_ capture technologies have to be developed for the implementation of CCS. Among various CO_2_ capture technologies, our institute focuses on membrane separation because transmembrane difference in concentration or chemical potential drives the separation and no additional energy is basically required. The smaller footprint of the facility, instant operation and lower maintenance cost are also important advantages in comparison to the current CO_2_ capture technology. Various CO_2_ separation membranes have been developed including organic, inorganic and hybrid membranes, such as mixed matrix membranes (MMMs) [6–8].

CO_2_ separation over H_2_ by membranes has been a topic for pre-combustion CO_2_ capture at an integrated gasification combined cycle (IGCC) plant, where the target gas consisted of mostly H_2_ and CO_2_ is pressurized, and the membrane separation is going to be suitable with organic or polymeric membranes. For the CO_2_ capture, CO_2_ is separated over smaller H_2_, and porous inorganic membranes and MMMs are not CO_2_ selective because the separation proceeds by molecular sieving mechanism. Polymeric membranes are dense and non-porous, and the gas permeability is explained by the following expression (Equation (1)). Ideal gas selectivity for gas *i* over *j α*_*i*/*j*_ is then given by the ratio of permeabilities as Equation (2).

(1) P=S×D

(2) $$a_{i/j}=P_{i}/P_{j}=S_{i}/S_{j}\times D_{i}/D_{j}$$

where *S* and *D* are solubility to the polymer matrix and diffusivity in the polymer, respectively [9]. Because the kinetic diameter of H_2_ (2.89 Å) is smaller than that of CO_2_ (3.30 Å), *D*(CO_2_)/*D*(H_2_) is smaller than one. For preferential CO_2_ permeation, *S*(CO_2_) should be much greater than *S*(H_2_). Amines have been incorporated physically or chemically into the polymeric membranes to enhance CO_2_ solubility in the membranes [10–15], where the amines also work as a CO_2_ carrier to facilitate the gas permeation through the membranes. PAMAM exhibits excellent CO_2_ separation performance [10,16], and the CO_2_ separation properties of PAMAM immobilized poly(ethylene glycol) (PEG) membranes have been extensively investigated in this research group [17–20]. The PAMAM membranes indeed exhibited excellent CO_2_ selectivity over H_2_, but they had a critical drawback. The CO_2_ permeability was not enough for the application, such as CO_2_ capture at the IGCC [21]. For the pre-combustion CO_2_ capture by membranes, CO_2_ permeance should be equal or above 100 GPU with 30 in the selectivity in a two-stage membrane separation process [21]. A plausible approach to elevate gas permeability is to reduce the membrane thickness. However, macrophase separation between the amines and PEG matrix on a couple of microns scale did not allow thinning the PEG membranes due to serious leakage of the physically entrapped amines from the matrix [18]. Herein, we described a solution to solve the above shortcoming with an alternate polymer matrix, PVA. PVA itself is known to have gas barrier nature and good membrane formability [22]. It also shows better compatibility to the amines and has been used as a membrane matrix for CO_2_ capture [11,13–15,21]. In addition, the CO_2_ separation properties of EA and MEA were compared with PAMAM in the polymer matrix. MEA is inexpensive and one of the most studied amines in liquid amine scrubbing [6,23,24]. Importance of the hydroxyl group was examined in the membrane separation.

## 2. Materials and methods

### 2.1. Materials

PAMAM (*G* 0, 20 wt% in methanol), PEG dimethacrylate (PEGDMA, MW 750), and 1-hydroxycyclohexyl phenylketone (Irgacure 184) were purchased from Sigma-Aldrich (Missouri, USA), and ethylamine (70 wt% in water) was from TCI (Tokyo, Japan). MEA and PVA (*M*_*w*_: 66,000, saponification: 85–90%) were obtained from Wako (Osaka, Japan). Other organic and inorganic chemicals were reagent grade and used without further purification. Gases with the highest purity grade commercially available were used for gas permeation experiment.

### 2.2. Membrane preparation process

For amine-containing PEG membranes, PEGDMA and amines were dissolved in ethanol, where the amine:PEGDMA:solvent ratio was kept to 6:4:5 by weight, and the monomer to initiator ratio was also kept by the molar ratio of 60. The reaction mixture was sandwiched with quartz plates with stainless spacers (50 μm in thickness) and exposed to UV light (365 nm, 12 mW/cm^2^, UVP B-100AP, CA, USA) for 3 min to polymerize PEGDMA [17]. The resulting polymeric membranes were dried under vacuum overnight. On the other hand, with amine-containing PVA membranes, 5 wt% of aqueous PVA was prepared by dissolving predetermined amount of PVA in deionized water at 90 °C overnight under stirring. Amines were then added to the aqueous PVA, where the weight fraction of them was varied from 10 to 90 wt% relative to the polymer. The resulting mixture was cast on a plastic petri dish and dried at ambient temperature for 2 days and then under vacuum at 40 °C for at least 6 h until no weight change was recorded. The membrane thickness was controlled by varying the casting amount and less than 50 μm. The membrane thickness was determined by a Mitutoyo digimatic micrometer (Tokyo, Japan).

### 2.3. Membrane characterizations

The thermal properties of amine-containing membranes were examined by differential scanning calorimetry (DSC) on a Netzsch DCS 204 F1 Phoenix (Netzsch, Kanagawa, Japan). 5–9 mg of the dried specimen was first cooled down to −100 °C, and thermal transitions upon heating were recorded with a heating rate of 10 °C/min from −100 to 250 °C. Because the amines were physically immobilized in the polymer matrices, they would be leached out from the polymer matrix in ethanol. The dried MEA- and PAMAM-containing membranes (ca. 120 mg) were dipped in 50 mL ethanol to remove the amine. The eluting solvent was changed every 12 h for 3 days. Because MEA was volatile under vacuum and heating, the eluted compounds in ethanol for PAMAM-containing membranes were recovered and determined by proton nuclear magnetic resonance (^1^H NMR) on a 600 MHz Avance III HD (Bruker, Kanagawa, Japan). Morphology of the resulting membrane was studied with a Hitachi S-4800 filed-emission scanning electron microscope (FE-SEM, Hitachi, Tokyo, Japan). The cross section of the membrane was prepared by freeze fracturing in liquid N_2_, and the obtained surface was coated with Pt before the SEM observation.

### 2.4. Gas permeation experiment

CO_2_ separation properties of amine-containing polymeric membranes were examined under isobaric conditions. Amin-containing polymeric membranes were placed on a porous PVDF support with nominal pore size of 0.45 μm (Durapore® membrane filter, HVLP02500, Merck Millipore) and set in a custom made flat sheet membrane cell [20], where the effective membrane area *A* was 2.27 × 10^−4^ m^2^. A CO_2_/H_2_ gas mixture (40/60 vol%) was humidified by passing through a water bath and fed to the membrane cell at a flow rate of 100 mL/min and 40 °C, and the CO_2_ partial pressure was 63 kPa in the operation conditions. Humidified He was supplied to collect the permeate at a flow rate of 10 mL/min and 40 °C. Gaseous species in both of the retentate and permeate were determined on an Agilent 7890A gas chromatograph equipped with a thermal conductivity detector and a pulse discharge helium ionization detector, respectively (Agilent Technologies, Tokyo, Japan). Gas permeability coefficient *P*_*i*_ and permeance *Q*_*i*_ were defined by the following equations.

(3) $$P_{i}=\frac{N_{i}\cdot l}{A\cdot t\cdot \Delta p_{i}}$$

(4) $$Q_{i}=\frac{P_{i}}{l}=\frac{N_{i}}{A\cdot t\cdot \Delta p_{i}}$$

where, *N*_*i*_, *l*, *t*, and Δ*p*_*i*_ were gas flux, membrane thickness, transmission time, and transmembrane pressure differences of gas *i*, respectively. The stage cut, a gas flux ratio of permeate/retentate, was controlled to less than 0.01, and under the experimental conditions, the pressure ratios of feed to permeate were sufficient to give the separation factor *α*(CO_2_/H_2_) as an ideal separation factor. The separation factor was expressed by Equation (3), where *x*_*i*_ and *y*_*i*_ were molar fraction of gas *i* in the feed and the permeate. The gas permeability coefficient and permeance were expressed in barrer and GPU, respectively; 1 barrer = 7.5 × 10^−18^ m^3^(STP)·m/(m^2^ s Pa) and 1 GPU = 7.5 × 10^−12^ m^3^(STP)/(m^2^ s Pa).

(5) $$\alpha_{i/j}=\frac{y_{i}/y_{j}}{x_{i}/y_{j}}≅\frac{P_{i}}{P_{j}}=\frac{Q_{i}}{Q_{j}}$$

## 3. Results and discussion

**Figure 1.**
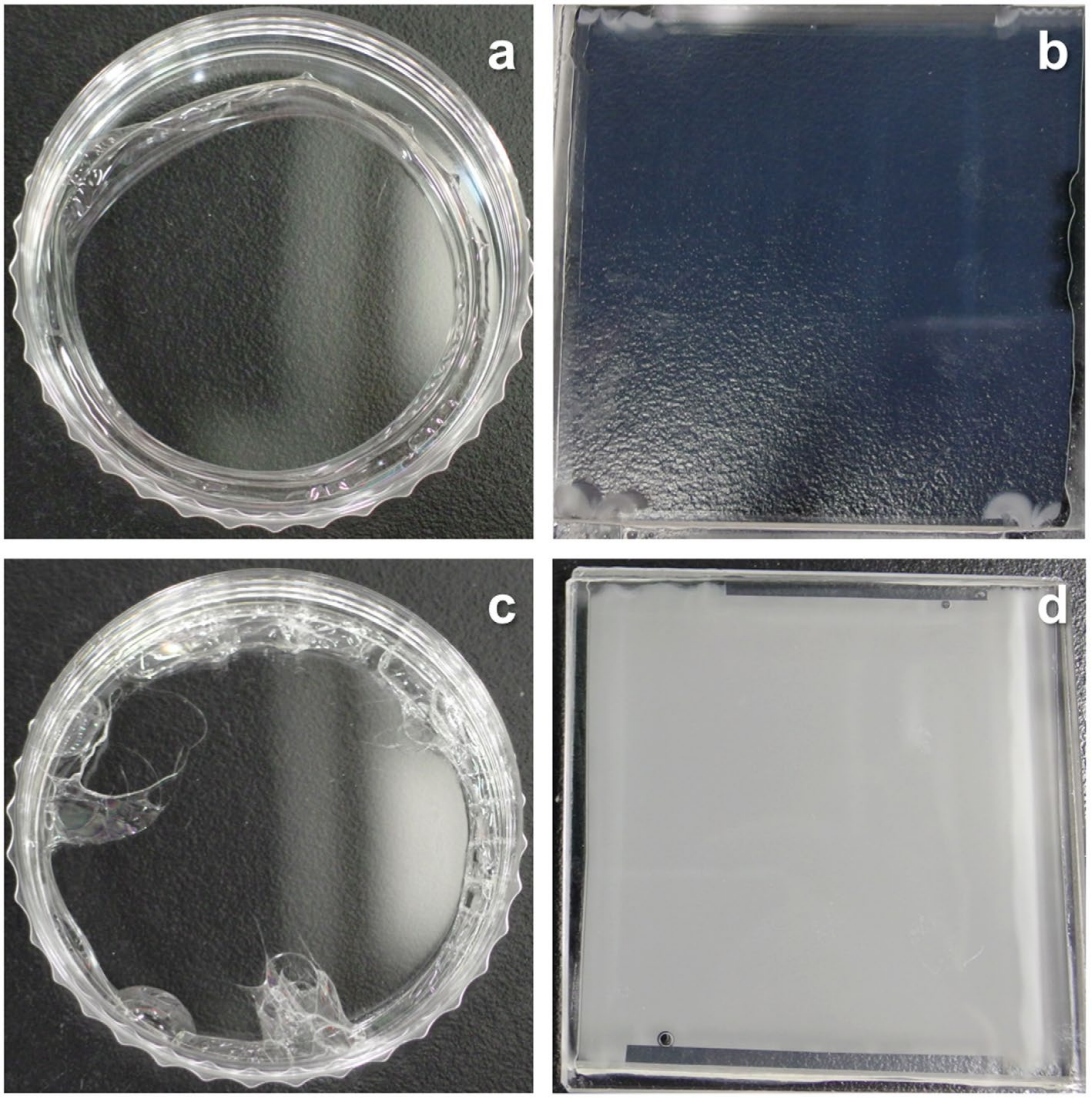
Optical images of (a) MEA-containing PVA, (b) MEA-containing PEG, (c) PAMAM-containing PVA and (d) PAMAM-containing PEG membranes.

CO_2_ separation by amine-containing polymeric membranes has been studied in this research group. Amines were readily immobilized in a cross-linked PEG by UV curing of PEGDMA in the presence of the amines [17]. However, poor compatibility between amines and PEG resulted in forming opaque or translucent membranes, which was due to macrophase separation on a couple of microns scale between amines and the matrix [18]. Thus, when the membrane thickness was reduced less than a few tens microns, leakage of amines were found. As a result, the membrane lost the CO_2_ separation performance. In comparison to the translucent PEG membranes, the amine-containing PVA membranes were transparent as shown in Figure 1. The better compatibility between amines and the matrix would reduce the phase separation.

**Figure 2.**
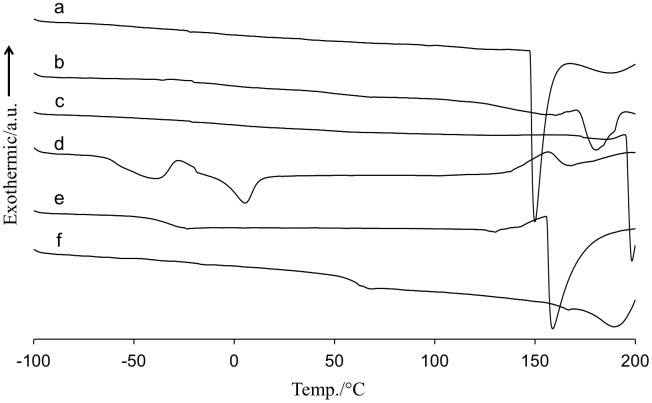
DSC thermographs of (a) MEA-containing PVA, (b) PAMAM-containing PVA, (c) EA-containing PVA, (d) MEA-containing PEG, (e) PAMAM-containing PEG and (f) pristine PVA membranes at heating rate of 10 °C/min.

One approach to examine the macrophase separation was expected to see thermal properties on DSC. The properties of the amine-containing PVA membranes would be different from pristine PVA due to change in the heat capacity upon phase mixing. Figure 2 displayed the DSC profiles of various amine-containing polymeric membranes. While pristine PVA membrane showed the glass transition temperature (*T*_*g*_) at 55 °C in Figure 2(e), the thermal transition of the amine-containing PVA membranes became ambiguous (Figure 2(a)–(c)). The glassy PVA became rubbery upon complexation with the amines. On the contrary, the amine-containing PEG membranes showed a clear thermal transition between −60 and −50 °C in Figure 2(d) and (e), which was corresponded to the *T*_*g*_ of PEG of the polymer matrix. Due to poor miscibility between the amines and PEG, a PEG-rich domain was formed, and the PEG matrix thus kept the inherent thermal properties even after the amine loading. An exothermic and endothermic peaks at −30 and 3 °C indicated crystallization and melting of MEA in Figure 2(d). The melting peak of PAMAM overlapped with the *T*_*g*_ of PEG and was not clear in Figure 2(e).

**Figure 3.**
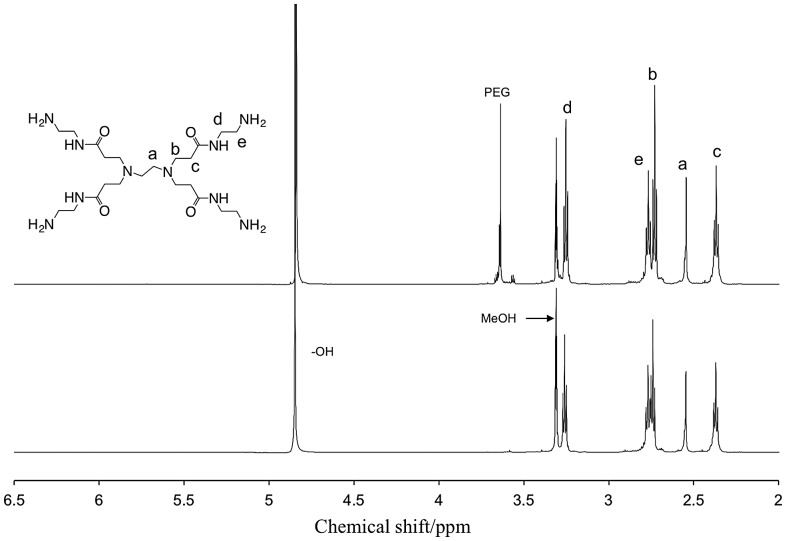
600 MHz ^1^H NMR spectra of residues in ethanol after amine elution test of (top) PAMAM-containing PEG and (bottom) PAMAM-containing PVA membranes in methanol-*d*_4_ at 25 °C, and the assignment of the peaks.

The macrophase separation was also studied by eluting out the incorporated amines in ethanol [25], which was a poor solvent for the polymer matrices. When the amine-containing PEG and PVA membranes were immersed in ethanol, physically immobilized amines came out into the solvent. For the PAMAM-containing membranes, the solutes were collected and determined as the incorporated amines in the matrices by ^1^H NMR as shown in Figure 3. This result suggested that the amines stayed intact in the polymer matrices even during the photopolymerization process to fabricate PEG membranes. In other words, Michael addition reaction between amines and vinyl compounds were negligible in the UV curing conditions. With the PAMAM-containing PEG membrane in Figure 3 (top spectrum), a peak was found at 3.6 ppm, which was the resonance of methylene protons of PEG. Oligomeric PEG would be released from the matrix. Because peaks of allyl protons of PEGDMA at 6.2–5.5 ppm were not seen, the monomer conversion would be completed.

On the other hand, with the MEA-containing membranes, MEA was found in ethanol by ^1^H NMR: 3.57 and 2.71 ppm for O–CH_2_ and N–CH_3_, respectively, in deuterated methanol. However, the amine was evaporated with ethanol during dry process, and the recovery was thus not precisely determined. In the PEG membranes, it was confirmed that the matrices were quantitatively recovered or physically entrapped amines were completely eluted out by the amine leaching experiment. On the contrary, while the amine fraction was 60 wt%, the weight reductions were 41 and 44% for the MEA- and PAMAM-containing PVA membranes. Better compatibility of the matrix to the amines suppressed the leakage from the membranes.

**Figure 4.**
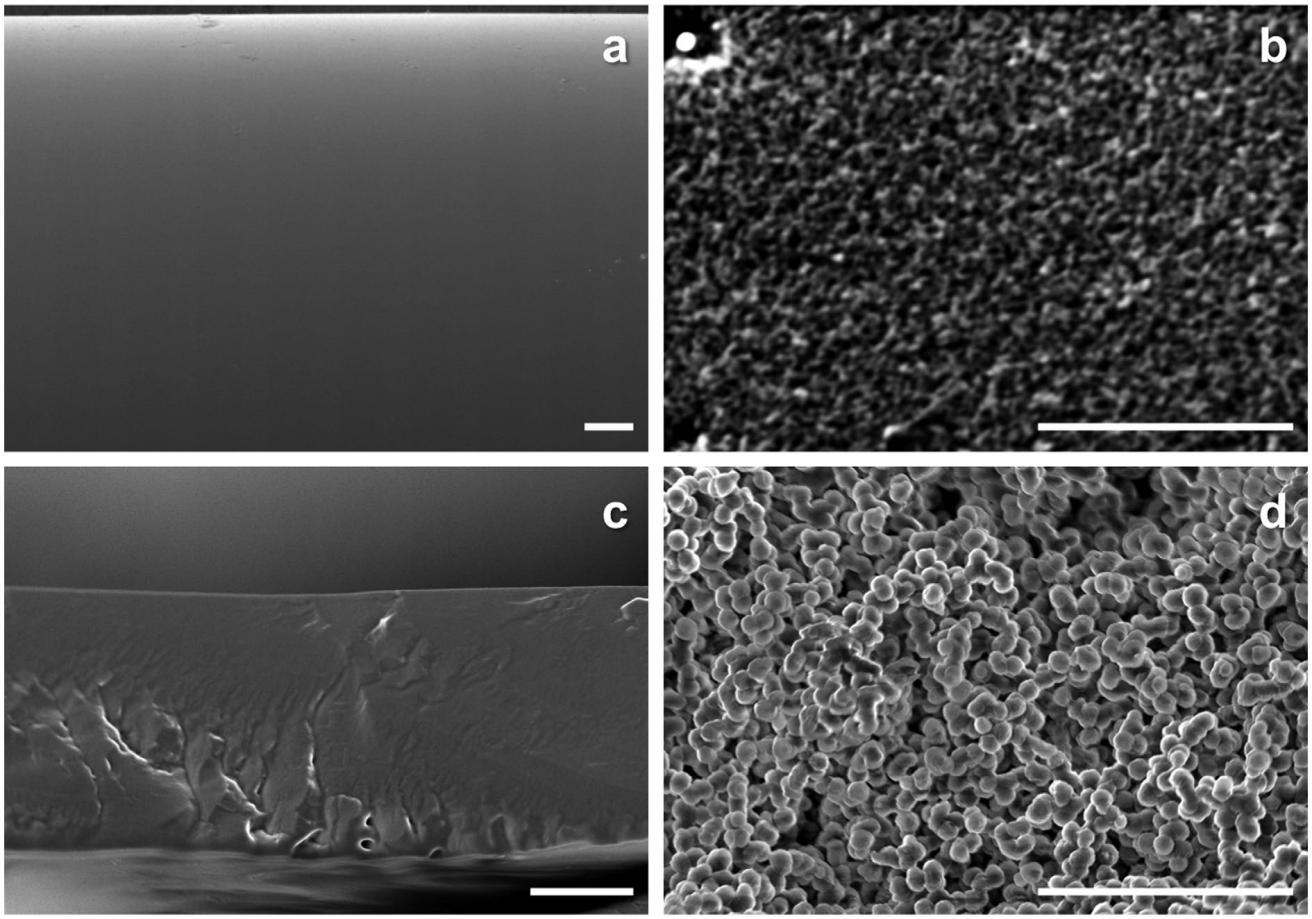
Cross-section SEM images of (a) MEA-containing PVA, (b) MEA-containing PEG, (c) PAMAM-containing PVA and (d) PAMAM-containing PEG membranes after amine elution test (bar: 10 μm).

The resulting matrices after the amine leaching were observed by SEM, and the obtained cross-section images were displayed in Figure 4. With the amine-containing PEG membranes, porous structures were found in the resulting matrix in Figure 4(b) and (d). The *T*_*g*_ of PEG was −60 °C and would be flexible during the amine leaching experiment at ambient conditions. However, cross-linking reaction confined the rearrangement of PEG chains, and the PEG-rich phase was fixed. As a result, the monolith like structure with micron-sized pores was remained, and the pore was originally filled with the amines immobilized in the UV curing of PEGDMA. The macrophase separated structures on a couple of microns scale resulted in the translucent membranes.

On the other hand, the amine-containing PVA membranes were transparent. Here, when the amines and PVA would be incompatible and have a similar photorefractive index, diffused reflection of light would be suppressed at the interface, and the resulting mixture would be transparent. In the case of the PVA membranes, quantitative recovery of the amines from the membranes was also confirmed after the ethanol immersion, and a flat and smooth surface was seen in Figure 4(a) and (b). When the entrapped amines formed the rich phase upon macrophase separation similar to the PEG membranes, a porous structure would be found because PVA was insoluble in the solvent and in a grassy state (*T*_*g*_: 55 °C in Figure 2(f)) in the experimental conditions. Thus, combined with the DSC results, it could be concluded that the amines and PVA were miscible on a micron scale, and a thinner membrane preparation was expected to be possible without amine leakage for the PVA matrix.

**Figure 5.**
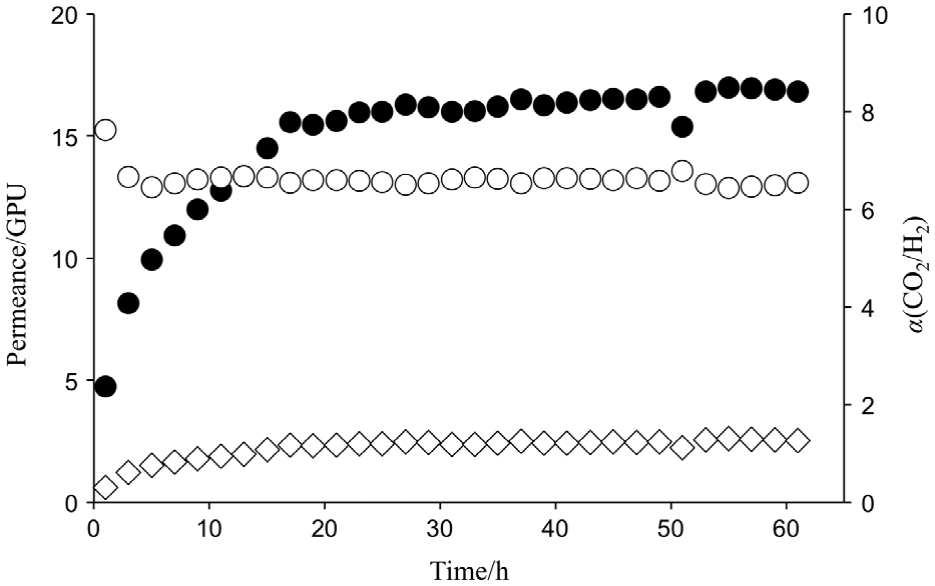
Change in gas transport properties of MEA-containing PVA membranes at 40 °C and 85% relative humidity (Δ*p*(CO_2_) =  63 kPa) as a function of time.

Note: The amine content was 50 wt%. Open circle: *Q*(CO_2_); open diamond: *Q*(H_2_) and filled circle: *α*(CO_2_/H_2_).

Gas permeation properties of amine-containing membranes were then investigated under isobaric conditions at 40 °C. The gas permeabilities changed with time by absorbing water under humidity and mostly reached equilibrium after 12 to 20 h incubation at the operation conditions as shown in Figure 5. Table 1 described a comparison of CO_2_ separation properties of MEA-containing PVA and PEG membranes over H_2_. The membrane thickness was measured immediately after the gas permeation test under humidified conditions. The PEG membranes were 52.7 μm in thickness, which was the minimum to prevent the amine leakage from the PEG matrix, and much thicker than the PVA ones (5.7 μm). The CO_2_ permeance of the PEG membranes was 7.9 GPU and lower than that of the PVA membranes (16.2 GPU). The higher CO_2_ selectivity of the PEG membranes was explained by higher amount of the amines, which worked as a CO_2_ carrier to facilitate the gas transportation through the membranes. On the other hand, the PVA membranes retained the CO_2_ separation properties over H_2_ by reducing the thickness to ca. 6 μm to give higher CO_2_ permeance.

**Table 1.** CO_2_ separation properties of MEA-containing PVA and PEG membranes over H_2_ at 40 °C and 90% relative humidity.

Matrix	CO_2_ permeance/GPU*	Selectivity	Thickness/μm
PVA	16.2	11.1	5.7
PEG	7.9	99.3	52.7

* 1 GPU = 7.5 × 10^−12^ m^3^(STP)/(m^2^ s Pa), [amine] = 60 wt%, Δ*p*(CO_2_) = 63 kPa.

**Figure 6.**
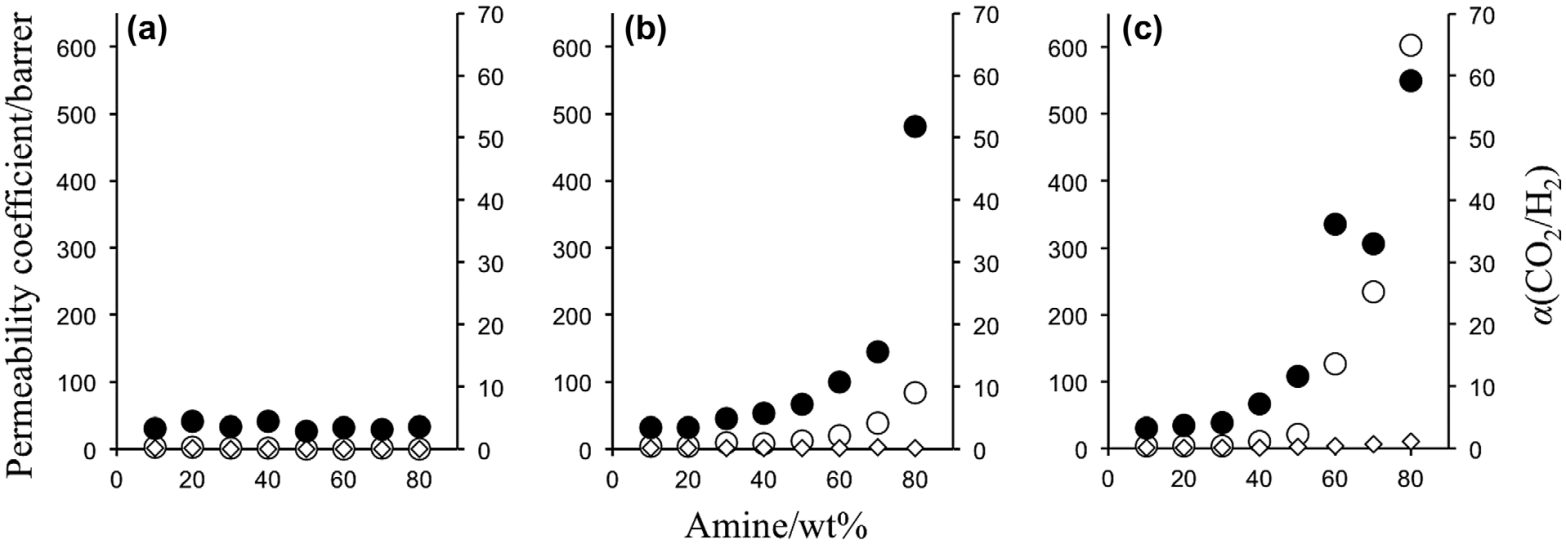
Effect of amine content on gas permeation properties of (a) EA- and (b) PAMAM and (c) MEA-containing PVA membranes at 40 °C and 80% relative humidity (Δ*p*(CO_2_) = 63 kPa).

Note: Open circle: *P*(CO_2_); open diamond: *P*(H_2_) and filled circle: *α*(CO_2_/H_2_).

In the PVA membrane preparation process, the amine content was varied from 10 to 90 wt% relative to PVA matrix, and self-standing membranes were obtained with the amine content of equal or less than 80 wt%. The amine leakage was negligible under the gas permeation operation conditions. Effect of amine loading on the CO_2_ transport properties was then investigated with self-standing membranes, and the results were shown in Figure 6. Because the membrane thicknesses of the specimens were from 3 to 43 μm depending on the amines and the contents, the gas permeability was expressed by permeability coefficient *P*, which was permeance normalized by the membrane thickness, 1 barrer corresponded to 1 GPU when the thickness was 1 μm.

**Figure 7.**
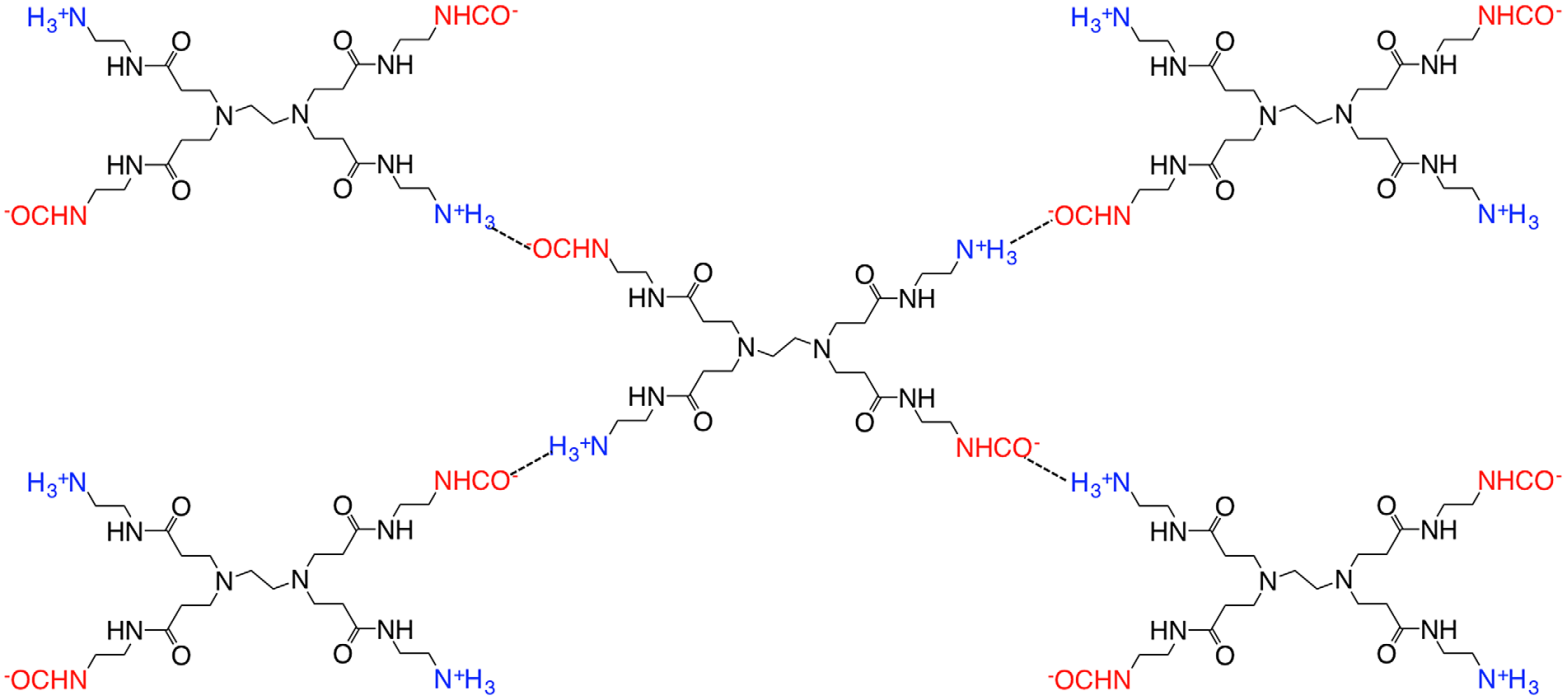
Schematic illustration of quasi-cross-linking of PAMAM with CO_2_.

For MEA- and PAMAM-containing PVA membranes, the CO_2_ permeance and separation factor increased with increase of the amine content, which indicated that the preferential CO_2_ permeation was based on facilitated transportation by the amines. The CO_2_ transport mechanism was different from those of the PEG membranes [17]. In the case of PAMAM-containing PEG membranes, the CO_2_ permeability slightly increased with increase of PAMAM content in the similar operation conditions. On the other hand, the H_2_ permeability significantly decreased, and as a result, the separation factor increased drastically with increase of the amine content. In the PEG membranes, the PAMAM-rich phase was formed upon microphase separation [18,25], and CO_2_ migrated the amine-rich phase rather than the PEG-rich phase due to difference in the CO_2_ solubility. When CO_2_ was fed to the membranes, dissolved CO_2_ reacted to the primary amines of PAMAM with the formation of carbamate as illustrated in Figure 7. PAMAM was crosslinked by the resulting carbamate ion pairs, and the quasi-crosslinks restricted the H_2_ permeation to provide a quite high CO_2_ selectivity by a CO_2_-selective molecular gate function [16,19].

On the contrary, the amines were compatible to PVA and did not form such amine-rich phase in the membranes confirmed by the SEM observations in Figure 4. The amines were diffused homogeneously through the PVA membranes and worked as a CO_2_ carrier to provide higher gas permeability, which was more preferable rather than the higher selectivity for practical CO_2_ capture [26].

$$2R-{\text{NH}}_{2}+\;{\text{CO}}_{2}⇄R-\text{NH}-{\text{COO}}^{-}+\;{\text{N}}^{+}{\text{H}}_{3}-R$$

Chemical structure of the amines was one of the crucial factors to characterize the CO_2_ separation performance in the PVA membranes. As depicted in Figure 6, MEA gave the highest CO_2_ permeation properties among the amines tested in the same weight fraction. With increase of MEA content from 10 to 80 wt%, the CO_2_ permeance and separation factor went up from 3.6 to 604 barrer and from 3.3 to 59, respectively. EA had the same carbon skeleton as MEA but no hydroxyl group, and the amine-containing membranes did not have effective CO_2_ separation performance. Thus, hydroxyl group of the alkanolamine would be a key to exhibit preferential CO_2_ permeation properties. The CO_2_ separation performance of the MEA-containing PVA membranes was actually higher than that of the PAMAM membranes. The CO_2_ permeability of the PAMAM membranes was less than 100 barrer and far below of that of the MEA membranes, while the separation factor was relatively smaller at the same amine weight fraction. A hydroxyl group in β-carbon adjacent to amino group helped to form a seven-membered ring when CO_2_ interacted to the amino group in the polymeric membranes [27,28]. The ring formation reduced the activation energy of the interaction and facilitated both of the association and dissociation between CO_2_ and the amino group, resulting in enhancing CO_2_ diffusion in the membranes.

## 4. Conclusions

The PAMAM-containing membranes have been studied for effective CO_2_ separation over H_2_. To reduce macrophase separation between the amine and the matrix, PVA was employed to improve compatibility to the amine. The resulting PVA membranes were transparent, and the DSC and the amine elution experiments clarified suppression of the macrophase separation. A thinner membrane formation was then possible with PVA matrix by reducing the phase separation, and an increase in CO_2_ permeability was confirmed. Furthermore, it was suggested that MEA exhibited higher CO_2_ permeability and selectivity than PAMAM in the polymer matrices. Hydroxyl group adjacent to the amino group would be a key to enhance the CO_2_ separation performance.

In this research, the CO_2_ separation properties over H_2_ was investigated under isobaric conditions, which was lower than the CO_2_ partial pressure of syngas after water-gas shift reaction at an IGCC. Thus, the separation performance under pressure was not clear. On the other hand, this membrane would hold potential to capture CO_2_ in the industrial H_2_ production process by steam reforming of light hydrocarbons. For example, H_2_ is generated from CH_4_ by the steam reforming followed by the shift reaction, and then purified over CO_2_ by pressure-swing adsorption (PSA). The PSA off-gas comes out at ambient pressure and contains certain amount of H_2_, which is used as fuel to keep the reformer at elevated temperature, and CO_2_ is in the off-gas eventually emitted. A feasibility study revealed that the CO_2_ separation properties observed could meet the required values to separate CO_2_ in the off-gas [29] and that the amine-containing PVA membranes would be applicable to make the current H_2_ process carbon-free.

## Disclosure statement

No potential conflict of interest was reported by the authors.

## Funding

This work was supported by Grant-in-Aid for Scientific Research (C) [grant number JP17899334]; the Advanced Carbon Technology Research and Development Program from Japan Science and Technology Agency; The International Institute for Carbon-Neutral Energy Research (WPI-I^2^CNER), World Premier International Research Center Initiative (WPI), MEXT, Japan.

## Supplemental data

Supplemental data for this article can be accessed at https://doi.org/10.1080/14686996.2017.1399045

## Supplementary Material

Supplemental_material.docx
