# Supplementary material for: Effect of amine structure on CO2 capture by polymeric membranes
Source: Sci Technol Adv Mater. 2017 Nov 22;18(1):950–8. doi: 10.1080/14686996.2017.1399045 (PMC5784313; doi:10.1080/14686996.2017.1399045)
Supplement: Supplemental_material.docx [file TSTA_A_1399045_SM3155.docx]

# Supplemental material

Effect of amine structure on CO_2_ capture by polymeric membranes

Ikuo Taniguchi^a,b^, Kae Kinugasa^a^, Mariko Toyoda^a^, & Koki Minezaki^b^

^a^International Institute for Carbon-Neutral Energy Research (WPI-I^2^CNER), Kyushu University, Fukuoka, Japan;

^b^Graduate School of Integrated Frontier Sciences, Kyushu University, Fukuoka, Japan

Corresponding author: Ikuo Taniguchi, Ph.D., International Institute for Carbon-Neutral Energy Research (WPI-I^2^CNER), Kyushu University, 744 Moto-oka, Nishi-ku, Fukuoka 819-0395, Japan; e-mail: ikuot@i2cner.kyushu-u.ac.jp

# FT-IR experiment

FT-IR measurements of polymeric membranes were carried out on a Shimadzu IR Prestige-21infrared spectrometer in Figure S1. The polymeric membranes were dried under vacuum, and the IR spectra were collected by accumulation of 30 scans with a 4 cm^-1^ slit at ambient conditions.


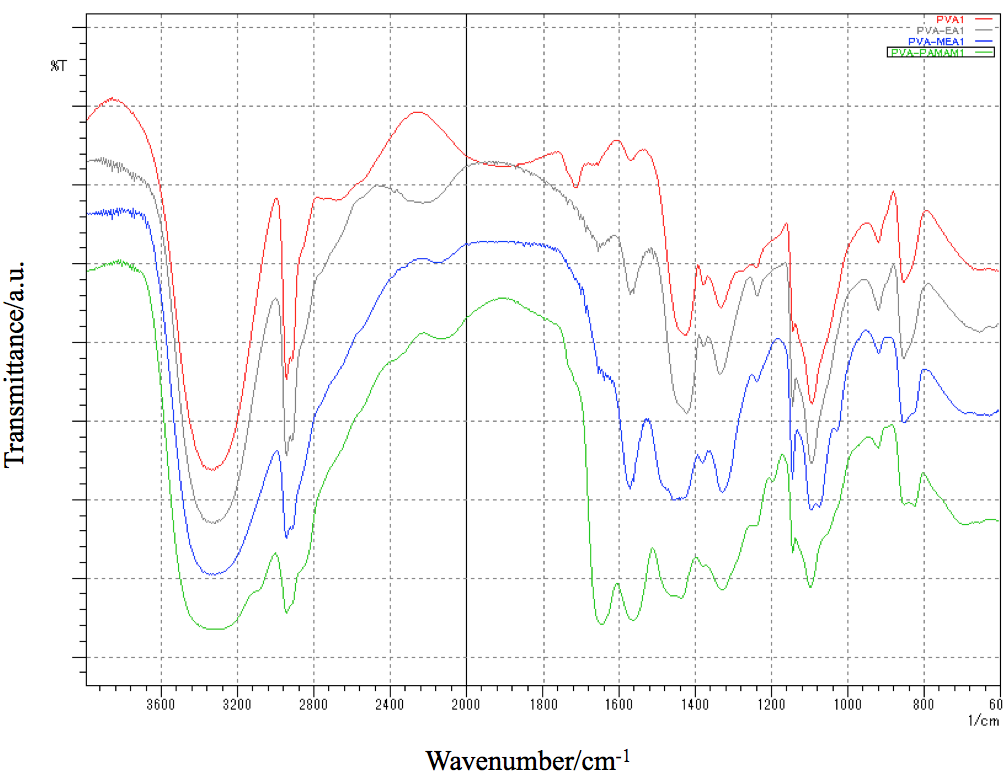


Figure S1. FT-IR spectra of EA-(grey), MEA-(blue), PAMAM-(green) containing and pristine PVA (red) membranes.
